# On-line information and support for supporters and carers of haematological cancer patients: is access an issue?

**DOI:** 10.1007/s00520-012-1388-9

**Published:** 2012-02-17

**Authors:** Christine L. Paul, Tara Clinton-McHarg, Marita Lynagh, Robert W. Sanson-Fisher, Flora Tzelepis

**Affiliations:** Priority Research Centre for Health Behaviour, University of Newcastle, University Drive, Callaghan, NSW 2308 Newcastle, Australia

**Keywords:** Haematological cancer, Support persons, Information, Support, Internet

## Abstract

**Purpose:**

This study aimed to assess levels of internet access, likelihood of using various sources of information or support, and sociodemographic characteristics related to high internet access among support persons of haematological cancer patients.

**Methods:**

A cross-sectional sample of haematological cancer survivors was recruited via a state cancer registry in Australia. Participating survivors invited their support persons to complete a survey. Of the 268 survivors, 68% had a support person return a survey. Approximately 80% of support persons reported having internet access.

**Results:**

Almost three quarters (74%) reported having ‘high’ access. Support persons reported their likelihood of using internet-based forms of information and support (59% and 26%, respectively) was lower than for other sources, including those delivered face-to-face (80% and 75%) or through print (87% and 70%). Participants who were older or had less education were less likely to report a high level of internet access or likelihood of using web-based sources.

**Conclusions:**

The results demonstrate the need to continue to provide information and support via multiple modes. Support persons who potentially are more vulnerable due to age and lower education are the least likely to use internet-based options. Consequently, these groups may require alternatives, including face-to-face or print-based information and support.

## Introduction

### High burden of psychosocial morbidity among people with haematological cancers

Cancer is an international health priority, with over 264,000 cases diagnosed in the UK, almost 1.6 million cases diagnosed in the USA, and over 100,000 new cases diagnosed in Australia each year [1–3]. In Australia, haematological cancers such as lymphoma and leukaemia represent the second largest cause of cancer mortality after lung cancer, as survival is poor compared to other cancer types [1]. Rates of clinical distress as measured by the Brief Symptom Inventory-18 can range from 32% to 48% for haematological patients given the debilitating nature of the disease and its treatment [4]. Diagnosis and treatment can also have a devastating impact on life expectancy, fertility and sexuality [5, 6], thus impacting on family functioning and relationships.

### Why is there an interest in support persons?

While little is known about those who support haematological patients specifically, carers of cancer patients more generally can report high levels of depression, anxiety and poor overall health [7–9]. Support persons also report a high need for information, practical support, assistance navigating the health care system and emotional or social support [10–12]. A recent Senate enquiry in Australia has also highlighted the issue of support for those caring for cancer patients [13]. However, it still appears that patient needs frequently take precedence over the needs of the caregiver [14].

### Challenges associated with delivering psychosocial care for support persons

There have been very few research trials which have tested effective interventions for delivering psychosocial support to carers of patients with haematological cancer. The majority of trials have investigated support provision for survivors only, but these have had limited success [14–16]. Potential reasons for the limited effectiveness of these interventions include poor uptake and patient preferences for self management [14, 15]. Given that individuals with carer responsibilities may have very little time available for seeking psychosocial support, effective support strategies are likely to be those which are highly accessible and available.

### The internet may be an effective vehicle for delivering psychosocial interventions to support persons

The internet offers unique advantages for the delivery of psychosocial interventions to cancer carers. First, it is widely accessible. Internet access has quadrupled between 1998 and 2008 [17]. The most recent Australian data suggests that 72% of the population has home internet access [17], while in the USA up to 69% of people have home internet access [18]. Second, the internet is already used by 77% of all cancer patients to access information about cancer, indicating high acceptability [19]. Third, for those in regional and remote areas [20], the internet overcomes some geographic barriers. It provides a way of connecting with information, services and others in a similar situation no matter their location without leaving home and at a time which suits their situation and level of wellness. It also offers the opportunity to provide peer on-line forums where support persons can obtain support from others in similar positions.

### Exploring internet access and intention touse among support persons of haematological cancer patients

While internet accessibility and use is apparently high among cancer patients, there is no current data about the accessibility or use of this resource for their support persons. Access to medical care often encompasses the dimensions of availability, accessibility, accommodation, affordability and acceptability [18, 19]. In the case of access to internet-based information and support, these dimensions can be conceptualised (as described in Table 1) as whether the individual has an internet connection that is: (1) Readily available for personal use (availability and affordability); (2) Allows information to be retained in a printed form (affordability and accessibility); (3) Is relatively free of connectivity problems (accessibility); (4) Is in a place that is comfortable and private (accommodation); and (5) Can be used with a high level of confidence to find information (acceptability). Therefore, identifying whether web-based approaches might be considered accessible for the provision of information and support to carers requires cumulative assessment of all the relevant dimensions.

**Table 1:** Survey items regarding internet access and preferred sources of information or support

Item	Response options
Level of internet access	
Accessibility	Yes, home; yes, work; yes; other, not at all
Do you have access to the internet?	
Accessibility	Available; most of the time, some of the time/rarely, no access
How would you describe your access to the internet for personal use?	
Connectivity	None, minor or occasional, major or frequent
Do you have any problems with access to the internet for personal use?	
Privacy	Very, moderately, not very
How private is the location where you use the internet for personal things?	
Comfort	Very, moderately, not very
How comfortable is the location where you usually use the internet for personal things?	
Printing	Yes as much as I like, yes—have to limit the amount, No
Are you able to print personal information from the internet?	
Confidence	Very, moderately , not very, never used
How confident are you in using the internet to find information?	
Likelihood of using sources of information and support	
Item 1: Often people who care for others with cancer need *information* (e.g. about cancer treatments, sources of financial help, or help with practical things like transport). If you needed this kind of information, how likely would you be to use the following sources?	
Item 2: Often people who care for others with cancer need *personal support* to cope with feeling down, stressed, anxious, or with trying to stay positive. If you were seeking this kind of help, how likely would you be to use the following types of support?	
Internet information	Very likely, likely, unsure, unlikely, very unlikely
Telephone information	
Printed materials	
Electronic media	
Face-to-face information	

In addition to access, it is important to estimate the likely uptake or use of internet-based information and support. Uptake rates of many support options by both patients and carers are quite low, for example, the helpline provided by the Cancer Council New South Wales in NSW receives only 16,000 calls per annum, which would equate to one call per 6.25 persons diagnosed with cancer in that year [21]. In order to assess the usefulness of developing support person-specific options for information and support, it is important to obtain an estimate of whether such options would be used by the target population.

### Socio-demographic factors may influence variations in accessibility and likelihood of use

Although reported levels of accessibility and use of the internet are high among cancer patients, differences in internet access can occur according to income, education, age and geographic location [17, 22]. Similarly, while internet access might be high for support persons as a group, it is important to identify whether access and potential uptake is uniformly high within subgroups of the support person population. Socio-demographic characteristics such as geographic location or age may affect access to this mode of support. For example, support persons living in rural locations may find internet-based options more appealing and accessible given their geographic isolation and reduced access to face-to-face resources. Conversely, older support persons may be less likely than their younger counterparts to have a high level of access to the internet or confidence in utilising web-based support. Therefore, an understanding of whether various information and support options (e.g. internet, face-to-face, printed materials, telephone support) might be used by either a large number of support persons, or by particular socio-demographic sub-groups, would be useful for guiding the investment of scarce resources into particular delivery modes of psychosocial support.

## Purpose

For a cross-sectional sample of support persons of haematological cancer patients, the study aimed to assess:

1. The proportion of support persons who report a high level of access to the internet. High level access is considered to be an internet connection which is frequently available for personal use, print-ready, rarely affected by connectivity problems, private and comfortable, and able to be used with confidence.
2. The proportion of support persons who report being likely to use various sources (internet, print, telephone, face-to-face) for information and support.
3. The socio-demographic characteristics of support persons who report: (1) a high level of internet access and (2) being likely to use the internet for information or support. Socio-demographic characteristics to be explored include: geographical location, age, gender, level of education, marital status, employment status, household size, social support and country of birth.

## Method

### Design and procedure

A registry-based approach to recruitment was used to permit sampling across the full range of haematological cancer types, locations and stages of treatment. One state cancer registry in Australia selected adult (aged 18–80 years at the time of the study) cancer survivors who had been diagnosed between 1 July 2007 and 30 June 2010 with leukaemia, lymphoma, or myeloma. Those who were deceased or who had previously indicated to the registry that they did not wish to participate in research were not eligible for the study. In total, 1,133 eligible patients were located in the registry. All rural patients were extracted to ensure sufficient representation from geographically isolated areas where internet-based approaches may be of benefit. All metropolitan patients were randomly sampled.

On behalf of the researchers, the Cancer Registry sent all eligible patients a information and questionnaire package for themselves along with a separate package to pass on to their primary support person. Results of the survivor survey are reported elsewhere. A support person was defined in the survey as “someone who has been significant to the cancer survivor on their cancer journey”. The support person questionnaire package contained an invitation letter, information statement, pre-paid envelope and a self-report pen and paper survey. Patients who did not respond to the initial questionnaire after 4 weeks were mailed a reminder letter from the Cancer Registry and a second questionnaire package, including a second questionnaire package for their primary support person. Reminders for support persons were not possible as their details were unknown to the researchers until they returned a survey. Return of a survey was taken as voluntary consent to participate in the study.

### Measure

The self-report pen and paper survey comprised a series of measures, a subset of which are reported here. The subset of survey items regarding information and support options was devised for the study and not previously tested for psychometric properties. The whole survey took an average of 30 min (SD = 13.7) to complete and included items regarding unmet needs, distress, depression, anxiety and stress , socio-demographic items, cancer type of the supported survivor, social support, financial and social impact of being a support person, views about being a support person and internet access. The items relevant here are:

#### Internet access and preferred sources of information or support

See Table 1 for a description of the survey items relating to internet access, use or preferences.

#### Socio demographic items

Items included gender, age, postcode (to assess rurality), marital status, Indigenous status, level of education, employment status, living arrangements (alone, with family, with friends), number of adults and children in household, country of birth and relationship to survivor and survivor diagnosis.

#### Social support

Items included whether or not there was someone s/he could confide in or discuss problems with.

### Analysis

Questions which were left blank or the response was incomprehensible were treated as missing data. An access score was calculated for each participant based on their responses to the level of internet access questions (Table 1). A high score consisted of five or more of the following responses: frequency of access (any/most time), connection problems (none/minor), privacy (moderately or very), comfort (very/moderately), printing (any or limited), confident (very/moderately). A moderate score was any three or four of these responses and low was 0–2. A score of none was given to those who indicated they had no access to the internet for personal use.

Logistic regressions were conducted to determine the factors associated with internet access and likelihood of use to aid in the support person role. High versus low or no internet access, the likelihood of using the internet for information (likely or very likely compared to unsure, not likely or very unlikely) and the likelihood of using the internet for support (likely or very likely compared to unsure, not likely or very unlikely) were entered separately as dependent variables. Initial χ^2^ analyses were conducted with the variables: living in a metropolitan area, gender, relationship to survivor, education, marital status, employment status, country of birth, household size, under 18 year olds in the household and availability of a confidant. Participants’ residential postcodes were used to classify their location as metropolitan or regional based on the Accessibility and Remoteness Index of Australia (ARIA+) classification. ARIA+ is the standard Australian Bureau of Statistics endorsed measure of remoteness, derived from measures of road distance between populated localities and service centres [23].

For this study, metropolitan was defined as those postcodes falling within the categories: major cities of Australia. Regional was defined as those postcodes falling within the categories: inner regional, outer regional, remote Australia, and very remote Australia. Age was analysed using *t* tests. Those variables with a *P* value less than 0.25 were included in a backwards stepwise logistic regression which removed variables until the model which minimised the Bayesian Information criterion was found. Analyses were conducted in Stata 11.1.

## Results

### Sample

A total of 732 eligible survivors (297 regional and 435 metropolitan) were invited to nominate their principal support person. Of these, 268 survivors (37%) completed a survey. Of the 268 participating survivors, 182 (68%) had a support person return a completed survey, with most support persons (83%) being partners of the cancer survivor. Table 2 presents the socio-demographic characteristics of the support person sample.

**Table 2:** Socio-demographic characteristics of sample (*n* = 181)

	Metropolitan		Regional		Total			
	*N*	%	*N*	%	*N*	%	Test	*P* value
*Age*	56.2	13.2	60.2	12.4	57.9	13.0	*F*(1,177) = 4.31	0.039
*Female*	75	73%	52	68%	128	71%	χ^2^(1) = 0.507	0.476
*Survivor diagnosis*								
Lymphoma	9	9%	4	5%	13	7%		
Leukaemia	29	29%	16	21%	45	25%		
Myeloma	16	16%	12	16%	28	16%		
NHL	46	46%	46	59%	92	52%	χ^2^(3) = 3.59	0.310
*Relationship to survivor*								
Partner	82	80%	67	87%	149	83%		
Relative	20	19%	10	13%	30	17%		
Other	1	1%	0	0%	1	1%	χ^2^(2) = 2.13	0.344
*Education*								
High school or less	44	43%	47	60%	91	51%		
Vocational training	21	21%	15	19%	36	20%		
University	37	36%	16	21%	53	29%	χ^2^(2) = 6.33	0.042
*Employed*	59	61%	33	44%	92	53%	χ^2^(1) = 4.81	0.028
*Married*	94	91%	74	95%	168	93%	χ^2^(1) = 0.868	0.352
*Australian born*	68	66%	55	71%	123	68%	χ^2^(1) = 0.412	0.521
TOTAL	103	57%	78	43%	181			

One participant could not be classified as metropolitan or regional due to a missing postcode and was left out of the demographic calculations. Not all categories add to 181 due to missing survey answers

#### Proportion with a high level of internet access

Of the 179 participants who answered the internet-related questions, 144 (80%) had home internet access and 38 (21%) had access at work. A minority (17%) reported having no internet access at all and an additional two (1%) had no access to the internet for personal use. Table 3 describes the nature of reported internet access among those who have access to the internet for personal use, indicating that approximately 74% of all participants have high levels of internet access. The most common constraints on internet access were lack of confidence with using the internet (57%) and minor or major problems with connectivity (40%). Residents in non-metropolitan areas were more likely to report connectivity problems (χ^2^(1) = 1.326, *p* < .003). No other significant differences in internet access were found between metropolitan and non-metropolitan internet users.

**Table 3:** Reported level of internet access

Nature of access	Proportion of those with internet access for personal use (*N* = 142)	
	*N*	%
*Frequency of access*		
Any	115	80
Most of time	24	17
*Connection problems*		
None	85	60
Minor	51	36
*Private*		
Very	95	67
Moderately	38	27
*Comfortable*		
Very	101	71
Moderately	38	27
*Can print personal info*		
Any amount	121	86
Limited amount	8	6
*Confident with internet*		
Very	61	43
Moderately	58	41
**Access score** ^**a**^	**Proportion of all respondents** (**N** = **175**)	
High	129	74
Moderate	10	6
Low	3	2
None	33	19

^a^See text for access score calculation

#### Likelihood of using various sources of information and support

As shown in Table 4, face-to-face and print are the preferred approaches for receiving both information and support. While over half of the sample reported they were likely to use the internet for information (59%), only a quarter were likely to use the internet as a medium for accessing support (26%). For both information seeking and receipt of support, the majority of the sample (89% and 72%, respectively) indicated they would use two or more sources. In the case of receipt of support, 19% indicated they were likely or very likely to use only one source of support, being predominantly face-to-face support (15% of respondents).

**Table 4:** Likelihood of using internet, telephone, print, electronic media or face-to-face forms of information and personal support (*n* = 175)

Source	Likelihood of using			
	For information		For Support	
	Likely/very likely to use		Likely/very likely to use	
	*N*	% (95%CI)	*N*	% (95%CI)
Internet	104	59 (52–67)%	45	26 (19–32)%
Telephone	95	54 (47–62)%	69	39 (32–47)%
Print	152	87 (82–92)%	122	70 (63–77)%
Electronic (DVD from Cancer Council, TV programmes, radio)	104	59 (52–67)%	87	50 (42–57)%
Face-to-face	140	80 (74–86)%	132	75 (69–82)%
**Number of options chosen as likely or very likely** ^**a**^				
None	5	3 (0–5)%	15	9 (4–13)%
Face-to-face only^a^	7	4 (1–7)%	26	15 (10–20)%
Print only^a^	4	2 (0–5)%	7	4 (1–7)%
One only	15	9 (4–13)%	34	19 (14–25)%
Two or more	155	89 (84–93)%	126	72 (65–79)%

^a^Likely/very likely for item of interest and not likely/very unlikely to all others

#### Socio-demographic characteristics of support persons with high levels of internet access and likelihood of using web-based sources of information and support

The logistic regression models indicated that a high internet access score was associated with a younger mean age and having a university degree compared to those without higher education (Table 5). These same groups were also more likely to report a being likely or very likely to use the internet to obtain information relevant to cancer. Younger participants were also more likely to report using the internet as a means of personal support.

**Table 5:** Final logistic regression models for support persons’ level of internet access and likelihood of using the internet for information and personal support

	High or likely mean (SD) or *N* (%)	Low or unlikely mean (SD) or *N* (%)	Odds ratio (95%CI)	*P*
*High access* (*n* = 147)				
Age	55.22 (12.49)	66.93 (8.22)	0.89 (0.84–0.94)	<0.001
Education				
School^a^	52 (71%)	21 (29%)		
Vocational training	22 (85%)	4 (15%)	1.62 (0.45–5.78)	0.459
University degree	45 (94%)	3 (6%)	6.52 (1.65–25.79)	0.008
*Likely to use the internet for information* (*n* = 155)				
Age	53.51 (12.24)	65.04 (10.04)	0.91 (0.87–0.94)	<0.001
Education				
School*	39 (52%)	36 (48%)		
Vocational training	20 (71%)	8 (29%)	1.68 (0.6-4.71)	0.325
University degree	39 (75%)	13 (25%)	2.64 (1.11–6.3)	0.029
*Likely to use the internet for support* (*n* = 155)				
Age	50.69 (12.46)	60.37 (11.85)	0.94 (0.91–0.97)	<0.001
Gender				
Male^a^	7 (14%)	43 (86%)		
Female	35 (33%)	70 (67%)	2.31 (0.9–5.95)	0.082

^a^Reference sample

## Discussion

This study is one of the first to document levels of access to and interest in internet-based information and support for cancer survivors.

### Is there a high level of access to the internet among support persons?

A high proportion of support persons (80%) reported having some internet access. Almost three quarters (74%) of the sample reported having a ‘high’ level of access in terms of frequency, privacy, comfort, print opportunity and confidence with internet use. As the latest reported general population figure is that of 72% of the Australian population having some form of internet access [17], support persons appear to have a similar level of access to that experienced by the population at large.

The 19% of respondents who reported being without any access to the internet for personal use suggests that a sizeable minority of support persons will continue to need alternative forms of access to information and support in the short to medium term. As might be expected, those with high access to the internet were more likely to be younger in age. Given that the mean age of the sample is high (mean age of 58 years), ‘older age’ indicates those in their 60s, 70s and 80s. It is also important to note that those with a university level of education were more than twice as likely as those with a school-only level of education to report a high level of internet access and likelihood of meeting information needs via the internet. Lower educational level is a well documented and important indicator of disadvantage [24] and is often linked to poorer health outcomes [25, 26]. Therefore, in order to avoid exacerbating health inequalities, healthcare providers and agencies that provide information and support should ensure that these remain available in non-internet formats which are appropriate to those of older age and lower education.

Among support persons who have some level of internet access, access constraints may include connection problems and lack of confidence. Minor connection problems were noted by 36% of those with access, with those in non-metropolitan areas more affected than those in metropolitan areas (47% and 28%, respectively). Only moderate levels of confidence were reported by 41% of those with internet access. The accessibility of internet platforms for the provision of information and support levels might be limited by these factors. Options for addressing these potential issues might include access to face-to-face or telephone-based training in internet use, as well as assistance with accessing internet information. This could be incorporated into appointment consultations during inpatient stays or patient seminars.

### What is the likelihood that support persons will use various sources of information and support?

The mode most likely to be used for both information and personal support was print (87% and 70%, respectively), followed by face-to-face (80% and 75%, respectively), with the internet being the least popular option (59% and 26%, respectively). The reported likelihood of using the internet for information (59%) was lower than the proportion who reported having internet access (80%), indicating that a sizeable proportion of those who use the internet for some forms of communication do not expect to use it as a source for cancer-related information. One hypothesis worthy of exploration is that this may, in part, be related to respondents’ views (not presented here) that some of the main disadvantages of the internet in relation to cancer information is a lack of personal specificity and uncertainty of information’s accuracy. As internet-based options may increasingly be able to provide both personalised information and personalised support, uptake may grow. Provider awareness can also be a potential barrier to patient utilisation of online cancer information [27]. An increase in provider awareness is important to enhance access to internet-based information sources. However, in the meantime the findings suggest alternative forms of information and support need to be maintained. This is particularly so for those who are older and do not have post-school education.

Most respondents reported that they would be likely to use two or more forms of information or support (89% and 72%, respectively). Few reported they would use only face to-face (3% and 15%), or solely print-based (2% and 4%) information or support. This indicates multiple sources of information and support is most likely to meet the needs of support persons. While this is not surprising, it does raise the dilemma of the most cost-efficient approach to providing information and support, particularly given the relative popularity and high cost of face-to-face options. Further exploration is needed to ensure face-to-face methods of information and support dissemination are indeed acceptable to support persons of cancer survivors, and who this information would be best received from. Face-to-face options such as group seminars and individual professional consultations should be explored.

#### Limitations

The generalisability of the study findings may be limited by the low response rate for the cancer survivors and therefore, limited opportunity for identifying support persons. While it is not possible to identify whether the socio-demographic characteristics of our sample could be considered representative of all support persons, the survivor sample was found to be reasonably representative in terms of gender, blood cancer type, and year of diagnosis (Hall A, Sanson-Fisher A, Lynagh M, Threlfall T, D’Este C, unpublished data). The reported likelihood of doing something is often higher than what happens in actual fact. This suggests people may overestimate their true likelihood of using any particular source. Therefore, the likelihood data should be interpreted in relative terms (e.g. likelihood of using print vs. internet) rather than absolute terms.

Low rates of expected use of the internet for support may have been in part due to difficulties in conceptualising how such support might operate. The question stem included examples: “e.g. online counselling programmes, online training in relaxation techniques, or online forums where you can talk to other cancer carers”. However, given the relative novelty of these approaches, it is possible than lack of familiarity may have influenced responses. Similarly, only brief explanations were provided regarding how each of the other proposed forms of support might operate. Therefore, the data may provide something of an underestimate of the popularity of some forms of support.

## Conclusions

There is a need to continue to provide information and support via multiple modes. Internet-based support while having a number of advantages, and accessible to most, is not embraced by all. Those who are potentially more vulnerable due older age and lower education are also those least likely to use internet-based options, and therefore require acceptable alternatives, which are most likely to be face-to-face or print based. While internet-based options hold promise, there is a need to improve their accessibility, perhaps via increasing familiarity for some and increased availability for others.
